# Brain tumor disparities: From biology to social determinants The 2019 Brain Tumor Epidemiology Consortium meeting report 

**DOI:** 10.5414/NP301234

**Published:** 2019-10-29

**Authors:** Kimberly J. Johnson, Luc Bauchet, Johannes A. Hainfellner, Carol Kruchko, Michael E. Scheurer, Joseph Wiemels, Judith Schwartzbaum

**Affiliations:** 1 Brown School Master of Public Health Program, Washington University, St. Louis, MO, USA,; 2 Montpellier University Hospital, Montpellier, France,; 3 Institute of Neurology, Medical University of Vienna, Austria,; 4 Central Brain Tumor Registry of the United States, Chicago, IL,; 5 Department of Pediatrics, Section of Hematology-Oncology, Baylor College of Medicine, Houston, TX,; 6 Department of Preventive Medicine, University of Southern California, Los Angeles, CA, and; 7 Division of Epidemiology, College of Public Health, Ohio State University, Columbus, OH, USA

**Keywords:** brain tumors, epidemiology

## Abstract

The Brain Tumor Epidemiology Consortium (BTEC) is an international consortium that fosters international and interdisciplinary collaborations focusing on research related to the etiology, outcomes, and prevention of brain tumors. The 20^th^ annual BTEC meeting with the theme “Brain tumor Disparities: From Biology to Social Determinants” was held in Los Angeles, CA, USA, on June 6 – 8, 2019. Scientists from the United States and Europe representing a broad range of brain tumor research disciplines presented their research findings at the meeting. The scientific content of the meeting is summarized below.

## Introduction 

The Brain Tumor Epidemiology Consortium (BTEC) provides a scientific forum for brain tumor epidemiology researchers conducting research related to improving our understanding of the etiology, outcomes, and prevention of brain tumors. In pursuit of its mission, BTEC members mentor junior and other investigators who are new to brain tumor epidemiologic research. BTEC was founded in 2003 following a meeting sponsored by the U.S. National Cancer Institute’s (NCI) Division of Cancer Epidemiology and Genetics (DCEG) and the U.S. National Institutes of Health’s (NIH) Office of Rare Diseases (ORD). BTEC has evolved to become a self-directed consortium whose members focus on the epidemiological aspects of brain tumors. Member research includes a wide range of topics pertaining to brain tumor etiology and outcomes and also includes methodological research related to classification and clinical risk prediction. BTEC is a U.S. National Cancer Institute-designated consortium and a nonprofit 501(c)(3) corporation. 

**Figure 1:**
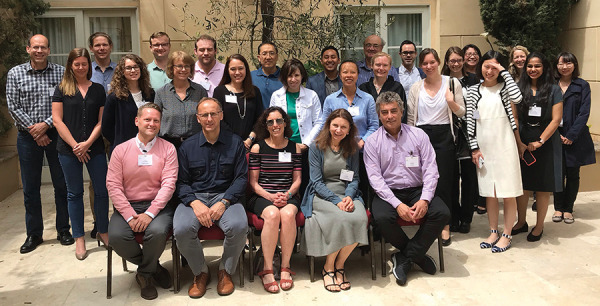
Attendees at the 2019 BTEC meeting in Los Angeles, CA, USA.

BTEC held its 2019 annual meeting in Los Angeles, CA, USA, with the theme *“Brain Tumor Disparities: From Biology to Social Determinants”*. The etiology of brain tumor development and survival is a complex process consisting of a combination of biological factors and social determinants. The program committee included Board of Director members: Co-Presidents Judith Schwartzbaum, PhD and Johannes Hainfellner, MD, PhD; Co-Vice Presidents Helle Broholm, PhD and Kim Johnson, MPH, PhD; Treasurer Michael Scheurer, PhD, MPH; and past Co-President Joseph Wiemels, PhD. The meeting was coordinated by Ms. Bénédicte Clement of Montpellier, France and Ms. Amy Yee of the University of Southern California. The meeting included three keynote addresses, five major presentations, and a panel discussion relevant to the meeting theme. In addition, there were 12 abstract presentations by junior and senior brain tumor researchers. Scientists from six countries representing a broad range of brain tumor research disciplines attended the meeting. A summary of the scientific content of the meeting is provided in the present report. A photograph of the 2019 BTEC meeting attendees is shown in Figure 1. 

## Summary of keynote lectures and major presentations 

**Kathleen Egan, PhD of Moffitt Cancer Center, Tampa, FL, USA, **gave a talk entitled *“Current evidence linking viral exposures to glioma risk”*. 

Dr. Egan provided background on the role of viruses in brain tumor etiology and described results from her research on human papillomavirus (HPV) and polyomaviruses (JC polyomavirus (JCV), BK polyomavirus (BKV), Merkel cell polyomavirus (MCV)) in glioma etiology. In two studies, no evidence was found for HPV DNA integration or expression. To study polyomaviruses in glioma etiology, Dr. Egan and her team procured biospecimens from the JANUS Biobank in Norway from 323 incident glioma cases and controls matched 1 : 1 to cases on age, gender, and blood collection year. Median time to glioma diagnosis from blood collection was ~ 15 years. No associations between JCV and BKV seropositivity or antibody titers and glioma were observed, which did not support suggestive findings from a prior study [1]. There was an increased odds of MCV seropositivity in glioma cases vs. controls, and the magnitude of association was equivalent in glioblastoma (GBM) and non-GBM tumors. In addition, there was suggestive evidence of a stronger risk associated with blood samples collected ≥ 20 years earlier and with greater antibody titer. She concluded her talk noting that the novel association between MCV and glioma that was identified needs confirmation in other study settings. 

**Luc Bauchet, MD, PhD of Montpellier University Hospital, Montpellier, France,** gave a two-part talk entitled *“The etiopathogenesis of diffuse low-grade gliomas (DLGG): the functional theory”* [2]. 

Dr. Bauchet reviewed the epidemiology and typical case presentation and noted that almost all DLGGs (defined as diffuse grade II glioma) progress to high-grade lesions, but both factors predicting progression and whether diffuse anaplastic gliomas (DAGs) come from DLGGs are unclear. Dr. Bauchet discussed DLGG management that is personalized for secondary prevention taking a short “wait-and-see” approach, followed by one or more surgeries first (when feasible), then chemotherapy, and radiotherapy last. Median survival can be very high, as long as 15 years. He concluded that for DLGG we still know very little because most studies do not specifically focus on this type of tumor. In the second part of his talk, Dr. Bauchet discussed the functional theory of brain tumor development, which asserts that certain tasks or training for these tasks may impact (positively or negatively) risk for tumor development through growth (and subsequent malignant transformation) of brain tissue associated with these tasks. Dr. Bauchet provided ancillary evidence in support of this theory from studies of jugglers and taxi drivers that show changes in gray matter and the posterior hippocampi, respectively, compared to their non-juggling and non-taxi driving counterparts [3, 4]. Moreover, mouse housing in enriched environment (with many toys) negatively modulates the growth of mouse and human intracranial glioma and significantly prolongs mice survival [5, 6]. Similar associations have not been studied in humans. 

**Reza Mirzaei, PhD of the University of Calgary, Canada,** gave a talk entitled *“T cell Exhaustion in Glioblastoma: Intricacies of Immune Checkpoints”*. 

Despite treatment advances, glioblastoma (GBM) survival time over recent decades has remained constant at ~ 30% of patients surviving 1 year after diagnosis, and mortality rates have also remain unchanged. Several features of GBMs lead to treatment failure in most patients, including: 1) tumor cell heterogeneity, 2) infiltration into the surrounding tissue, 3) location in the blood-brain-barrier refuge, and 4) suppression of an antitumor immune response. The latter is being actively pursued as a therapeutic leverage point. Dr. Mirzaei reviewed principles underlying PD-1/PD-L1 antibody blockade immune therapies that capitalize on the interaction between expression of PD-1 on cytotoxic T-cells and PD-L1 on tumor cells. The interaction between these molecules sets up a scenario favoring tumor cell survival and immune cell death. PD-1/PD-L1 therapy has a high objective response rate for some cancers but has not shown efficacy for GBM in clinical trials. Key reasons underlying this lack of response are thought to include the lower mutational load in GBM cells making them less antigenic to cytotoxic T-cells, a lower T-cell density in GBM tumors, the blood brain barrier that impedes therapeutic antibodies from migrating into the brain, and the presence of macrophages (microglia) in the tumors that aid tumor cell survival through anti-PD-1 antibody uptake. Further complicating the efficacy of immune system therapeutic approaches is immunosuppression in GBM patients due to treatment with steroids, temozolomide, and radiation therapy. Another challenge is the metabolic profile of the GBM microenvironment where 25% of glucose is devoted to the brain; GBM cells are highly glucose dependent, resulting in vigorous competition for glucose between anti-tumorigenic immune cells that are also glucose dependent and glial cells. Dr. Mirzaei in Dr. Wee Yong’s lab is actively investigating the roles of PD-1/PD-L1 in glucose competition within the GBM environment with the goal of developing therapeutic strategies that target glucose metabolism. 

**Adelheid Woehrer, MD, PhD of the University of Vienna, Austria,** gave a presentation entitled *“The DNA methylation landscape of glioblastoma disease progression shows extensive heterogeneity in time and space”*. 

Understanding the epigenome could open up new preventive and therapeutic opportunities as the number of drugs targeting the epigenome is increasing. Dr. Woehrer presented results from two studies focusing on epigenomic aspects of GBM. The first study showed the feasibility of using archival formalin-fixed paraffin-embedded tissues for epigenomic profiling and identification of GBM biomarkers, while the second study demonstrated that DNA methylation subtypes generally remain stable over time, clonality dictates temporal sequence, and tumor evolution is influenced by treatment [7, 8]. Dr. Woehrer also described the Glioma Longitudinal Analysis Consortium (GLASS), a community-driven effort with 19 participating institutions and The Cancer Genome Atlas (TCGA) that has been assembled to overcome significant challenges in constructing datasets with longitudinal measurements. She explained that longitudinal cohorts combining multiple molecular platforms are needed to provide relevant new insights into how the epigenome plus the genome as well as immunological and metabolic background shape tumor evolution. She also noted the need for incorporation of longitudinal samples into clinical trial design and routine patient management. Finally, she described how an important future direction will be to establish links between molecular and phenotypic data such as fMRI imaging. Her preliminary data on tumor-related impacts on brain networks shows potential utility for early detection of tumor recurrence. 

**John Wiencke, PhD and Annette Molinaro, MA, PhD of the University of California San Francisco (UCSF), San Francisco, CA, USA,** gave a joint talk entitled *“Blood immunomethylomics as a marker of glioma outcomes at UCSF”*. 

Dr. Wiencke gave an overview of the UCSF Immune Profiles program that has the goals of identifying immune profiles in blood associated with glioma outcomes and of evaluating associations between blood immune profiles and tumor genetic and immune characteristics. He provided a definition of immunomethylomics as a DNA-based method for quantifying immune cell subsets based on subset-specific DNA methylation signatures. He noted that the UCSF group is exploring two research questions using immunomethylomics: 1) Do peripheral immune profiles predict recurrence and survival in lower-grade gliomas and GBM? 2) What are the most important elements of the immune profile for patient management? To develop models that predict groups by survival, his group employs a decision-tree analysis statistical technique called partitioning using deletion, substitution, and addition (PartDSA). Dr. Molinaro showed preliminary data using PartDSA applied to retrospective samples and shared their plan for collecting and analyzing prospectively collected samples. Dr. Molinaro concluded their talk with four points: 1) immunomethylomic-based analysis provides a powerful method for assessing immune profiles prospectively and from archived blood specimens that are not amenable to flow cytometry, 2) cross-sectional data of peripheral whole blood from several molecular subtypes of glioma demonstrates that immune cell proportions were associated with survival, 3) recursive partitioning via the partDSA survival models showed neutrophils, CD4, CD8, and total lymphocyte proportions distinguished survival groups more strongly than grade, and 4) prospective, longitudinal collection of peripheral blood from glioma patients is feasible, and assessment of immune profiles with immunomethylomics will provide further insight into the role of systemic immune profiles over the disease course. 

**Panel discussion: “Is glioma caused by “bad luck”? If so, is further research on glioma etiology productive?”**

The panel discussion was stimulated by a controversial 2015 paper entitled “Variation in cancer risk among tissues can be explained by the number of stem cell divisions” [9]. Dr. Schwartzbaum reviewed the paper where a major take away was that many cancers arise through unavoidable bad luck and that research should focus on early detection, rather than etiology or prevention. She pointed out that the conclusions in this paper are based on the authors’ misunderstanding of the meaning of r^2^. The panelists (Drs. Egan and Bauchet) were asked to discuss their perspectives on whether glioma is largely due to bad luck. Dr. Egan posed some thought-provoking questions and facts that contradict the bad luck theory for glioma: 1) Why is glioma more common in men? 2) Why is it more common in U.S. Whites than Blacks? 3) How do we explain the geographic variation within the U.S./Europe? 4) Why are migrants to the U.S./Canada at differential risk when compared to children born to them? 5) If genetic variants explain at most ~ 27% and ~ 37% of familial risk of GBM and non-GBM, respectively; what explains the rest, and could it be shared environments? 6) as ionizing radiation exposure is an established risk factor; should we be concerned about cumulative exposure to medical ionizing radiation?, and 7) we already know that telomere biology plays a major role in glioma risk, and extensive physiologic, genetic, and environmental factors impact telomere biology. Dr. Luc Bauchet provided a clinician point of view and made the important point that for the patient, the cause of glioma is always “bad luck”. He also pointed out that patients and their relatives always want to know why, whether it was inevitable, and what can they do outside of treatment to slow down tumor development. He emphasized that clinicians need help from epidemiologists to answer these questions. He ended with a rhetorical question and answer that even if glioma arises through bad luck, is prevention impossible? After answering “no”, he explained that secondary prevention (screening) may be possible for diffuse low-grade glioma [10]. He noted, however, there is the question of what to do with incidental findings that may be detected in screening and noted there is stress caused by a wait-and-see approach that needs to be factored into the discussion. 

**Daniel Notterman, MD, MA of Princeton University, Princeton, NJ, USA,** gave a talk entitled *“Epigenetics and Social Determinants of Health”*. 

Dr. Notterman presented an overview of epigenetics and included several examples providing evidence that a person’s epigenome changes with time and exposure to environmental factors. Identical twins at the age of 3 years have almost the same methylation states. However, he noted that after several decades their methylation patterns will be different, which is mainly attributable to environmental signals as well as normal aging. He also discussed several Dutch Famine studies showing that in utero conditions can affect epigenetic patterns and outcomes [11, 12, 13, 14]. The last part of his talk focused on his research in the Fragile Families and Child Wellbeing study, a longitudinal study of families including ~ 5,000 children born to predominantly unmarried mothers that aims to understand what makes these families at particularly high risk of poverty and other challenges. The study includes extensive surveys of children, mothers, fathers, primary caregivers, child-care providers, teachers, and biological measurements. Part of this study investigates how the challenges that these families face affect the epigenome as well as telomere length. Dr. Notterman showed some preliminary data providing evidence that changes in DNA methylation can reflect some of the effects of social disparities on health. 

**Maria Feychting, PhD of the Karolinska Institute in Stockholm, Sweden,** presented a talk entitled *“Socioeconomic disparities in brain tumor risk and survival”*. 

Typical indicators of socioeconomic status (SES) used in research include education, income, occupation, and marital status. Dr. Feychting presented some examples of research on SES and brain tumors from the U.S. [15, 16, 17, 18, 19, 20, 21], and concluded that most studies not relying on subject participation report higher glioma incidence associated with higher SES. She noted several possible explanations including high SES as an indicator of environmental risk factors, detection bias, healthcare seeking behavior, and confounding of SES by comorbidities. In the second part of her talk, Dr. Feychting gave an overview of research on SES differences in survival after glioma diagnosis and noted that several countries show consistently poorer survival after glioma diagnosis among patients with lower SES. She noted that even in Sweden, where there is universal healthcare, there is evidence for an increased hazard of death for glioma and meningioma patients associated with measures of SES [19]. Potential explanations of these findings include correlations between lower SES and poor health, and patient and doctor diagnosis delays. 

**Michael Scheurer, PhD of the Baylor College of Medicine, Houston, TX, USA,** gave a talk entitled *“Social determinants of disparities in risk and outcomes”*. 

He started with a definition of the social determinants of health as conditions in the environments in which people are born, live, learn, work, play, worship, and age that affect a wide range of health, functioning, and quality-of-life outcomes and risks. He reviewed the U.S. Centers for Disease Control and Prevention (CDC) Healthy People 2020 five Key Areas of social determinants of health that include: 1) Economic stability, 2) Education, 3) Health and Healthcare, 4) Neighborhood and Built Environment, and 5) Social and Community Context [22]. He also gave an overview of the Area Deprivation Index (ADI) that combines 17 different indicators of income, education, employment, and housing conditions at the census tract level to show where areas of deprivation or affluence exist in communities. The ADI can be used as a social exposure indicator with a low ADI being linked to several adverse outcomes including cancer. Dr. Scheurer provided data on incidence trends of childhood brain tumors diagnosed at ages 0 – 14 years by several measures of SES available in Surveillance, Epidemiology, and End Results data. Finally, Dr. Scheurer ended with a conceptual model for potential pathways of the effects of social inequalities on brain tumor risk [23]. 

## Abstract presentations 

There were 12 abstract presentations that covered topics including the descriptive epidemiology of brain tumors, risk factors, access to care, and statistical methodology pertaining to brain tumor research. The first abstract session began with two Young Investigator Award presentations. The first presentation was given by **Friederike Erdmann, PhD, MPH of the Childhood Cancer Research Group of the Danish Cancer Society Research Center, Copenhagen, Denmark**. The talk was entitled *“Are tumors of the central nervous system in children associated with socioeconomic factors? Findings from a nationwide case-control study in Denmark”*. 

Dr. Erdmann described the results of a Danish nationwide, matched case-control study of central nervous system (CNS) tumors diagnosed before age 20 years that aimed to: 1) evaluate SES differences in the risk of childhood CNS tumors, and 2) examine whether associations differ by CNS subtype. Measures of SES were identified from nationwide registries and included parental highest attained education and disposable income. Among 6,359 cases and controls, a slightly increased risk of CNS tumors in association with higher maternal education was observed for all three time points under study (at conception, during pregnancy, and 1 year before diagnosis). Similar findings were observed for paternal education at time of 1 year before diagnosis but null associations for paternal education before and during pregnancy. With respect to specific subtypes, associations were stronger but less precise. Risk also increased with increasing maternal disposable income before diagnosis overall and by subtype (embryonal, and astrocytoma and gliomas). Dr. Erdmann ended with a discussion of underlying mechanisms and noted that under-ascertainment is unlikely because of high-quality healthcare services in Denmark. 

The second Young Investigator Award presentation entitled *“Immune factors preceding diagnosis of glioma, a Prostate Lung Colorectal Ovarian Cancer Trial nested case-control study”* was given by **Ivo S. Muskens, MD, of the University of Southern California, Los Angeles, CA, USA**. 

Dr. Muskens described results from a nested case-control study using data from the Prostate, Lung, Colorectal, and Ovarian (PCLO) randomized controlled screening trial that aimed to identify glioma risk factors. Using conditional logistic regression, Dr. Muskens examined risk of glioma in association with weight, height, comorbidities, family history of cancer, and immune factors (total IgE, immune specific IgE, IL13, TGF-β1, TNF-α). In addition, he employed a time-dependent Cox model for immune factors for individuals with follow-up samples. The study included 124 cases and 496 controls. Height and BMI were not statistically significantly associated with glioma. Family history of glioma was identified as a risk factor. For immune factors, elevated immune-specific IgE was associated with a decreased risk of glioma. TGF-β was associated with an increased risk, and IL13 was associated with a decreased risk in time-dependent Cox models. Overall, the results suggested that immune factors play a role in brain tumor etiology. Dr. Muskens ended with some clinical implications, including prediagnostic screening, and identification of patients who might be responsive to anti-TGF-β therapy. 

**David J. Cote, MD/PhD student at Harvard Medical School and Harvard T.H. Chan School of Public Health, Boston, MA, USA**, winner of a travel award for his abstract, presented a talk entitled *“Glioma incidence and survival variation by county-level socioeconomic measures”*. 

Mr. Cote presented results from a study using data from the Central Brain Tumor Registry of the United States (CBTRUS) from 2011 – 2015 and survival data from SEER 2000 – 2015. Measures included urbanicity, defined by Rural Urban Continuation Codes, and a county-level socioeconomic status (SES) variable constructed from the 2010 American Community survey. Key findings were higher glioma incidence rates in counties with higher SES for white non-Hispanic individuals, but not other ethnic groups. With regard to survival, individuals in high SES vs. low SES counties exhibited improved survival, and white non-Hispanic individuals exhibited worse survival than other racial and ethnic groups. Mr. Cote and coauthors concluded that differences in both incidence and survival were largely driven by race and SES rather than by urbanicity. 

The next abstract talk entitled *“Comprehensive review of cranial chordomas using national databases in the United States”* was given by **John L. Villano, MD, PhD of the University of Kentucky in Lexington, KY, USA**. 

Dr. Villano and his team conducted a descriptive epidemiology study of cranial chordomas that originate from remnants of the notochord along the neural axis. These tumors are diagnosed in < 300 individuals a year in the United States. Using data from the National Cancer Database (NCDB) 2004 – 2014, SEER 2004 – 2014, and National Surgical Quality Improvement Program NSQIP from 2005 – 2016, Dr. Villano reported that the use of surgery and radiation is increasing with a trend towards longer survival. The results also demonstrated that 30% of patients receive proton therapy, and its use has increased in recent years, craniotomies for chordoma resection have a longer operative time, hospital stay, and a higher rate of complications, and radiation modality type did not statistically impact survival. 

**Qianxi Feng, MPH, a doctoral student at the University of Southern California, Los Angeles, CA, USA,** described the results of a study using Surveillance, Epidemiology, and End Results 18 data entitled *“Incidence and survival of pilocytic astrocytomas in children in the USA, 2000 – 2016”*. 

The study included patients diagnosed from 2000 – 2016 with pilocytic astrocytomas at the ages of 0 – 19 years. A key finding included the observation that the highest age-adjusted incidence rate occurred among non-Latino Whites, followed by non-Latino American Indian/Alaskan Natives, Latinos, and non-Latino Blacks. Among cases diagnosed from 2000 – 2011 who had surgery, the 5-year survival rate was lowest among non-Latino Blacks; however, there was no difference in 5-year survival after adjustment. Year of diagnosis and radiation and chemotherapy were significant predictors of mortality in pilocytic astrocytoma. 

**Stephen Francis, PhD of the University of California at San Francisco, San Francisco, CA, USA,** gave a talk entitled *“Socioeconomic Status and Childhood Central Nervous System Tumors in California“*. 

Using California cancer and birth registry data, Dr. Francis and colleagues conducted a case-control study including 3,022 cases with a primary CNS tumor diagnosed at ages 0 – 19 years and 10,791 matched controls. SES measures included birth certificate-reported parental education and insurance utilization as well as census tract level median household income and percent of adults not completing high school. Associations between markers of SES and CNS tumors were estimated using conditional logistic regression adjusting for known or suspected risk factors. Using both types of SES measures, low SES was associated with a reduced risk of childhood CNS tumors. 

**Xiwen Huang, MPH** gave a talk on behalf of **Julia Heck, PhD of the University of California Los Angeles, Los Angeles, CA, USA,** entitled *“Hepatitis in Taiwan and risk for pediatric CNS tumors”*. 

This study linked birth records to the Taiwan Cancer Registry and National Health Insurance database (NHID) and included all children born in Taiwan 2004 – 2014. A total of 260 children with CNS cancer were included with maternal hepatitis during pregnancy ascertained from the NHID. Using unconditional logistic regression, no association between CNS tumors and hepatitis B (OR = 1.06, 95% CI 0.63 – 1.79) was found; however, the odds of a maternal hepatitis C history was 3.21 (95% CI 1.43 – 7.22) times higher in cases than controls. 

**Pavan Shrestha of the University of California San Francisco, San Francisco, CA, USA,** gave a talk entitled *“Recruitment and longitudinal sample collection in an immune profiles study in adult glioma patients”*. 

He described the overall workflow for a study that aims to determine 1) whether immune profiles predict recurrence and survival in lower-grade gliomas and glioblastomas and 2) the most important immune profile elements for patient management. He discussed pathways of recruitment and described REDCap as a useful database for tracking data and sample information. He concluded from their experience thus far that it is feasible to collect longitudinal blood samples from glioma patients at UCSF and noted that keys to success include identifying eligible patients through multiple methods, having multiple blood draws, maintaining close communication between staff, timely follow-up reminders, close patient tracking, and meticulous record keeping. 

**Helen Hansen of the University of California San Francisco, San Francisco, CA, USA,** gave a talk entitled *“Applying quantitative immunomethylomics to evaluate peripheral T-cell status among glioma patients before and after surgical resection”*. 

She reported her group’s preliminary results from a study that has the goal of longitudinally assessing blood immune profiles in glioma patients before and after treatment using immunomethylomics. Using 72 patient samples, the results to date demonstrated that quantitative immunomethylomic counts of CD4T cells derived from frozen whole blood samples are in good agreement with clinical flow cytometry measurements. She also reported the observation that low CD4T cell counts in GBM and recurrent low-grade glioma were found prior to intervention with surgery, radiation, and chemotherapy (TMZ). Future directions include tracking the trajectory of peripheral immune factors in glioma patients over the course of their treatment and assessing whether specific immune profiles predict patient outcomes. 

**Yalan Zhang, MS of the University of California San Francisco, San Francisco, CA, USA,** gave a talk entitled *“Implementation of a targeted next-generation sequencing panel for the diagnosis and precision medicine treatment of adult patients with WHO grade IV gliomas”*. 

Using the UCSF500NGS panel, the utility of a curated cancer gene sequencing panel, for predicting outcomes and matching patients to therapy based on their tumor genetic profile, was tested. Ms. Zhang gave two case examples demonstrating the clinical utility of the panel. She concluded that routine genomic profiling in adult GBM results in identification of tumors with molecular profiles that negatively impact prognosis, potentially actionable genetic alterations, previously unknown germline mutations, and a subset of glioblastomas (~ 4%) that harbor somatic hypermutations that may be amenable to immune therapy. 

The last abstract talk was given by **Pranathi Chunduru of the University of California San Francisco, San Francisco, CA, USA,** entitled *“Multiparametric MR-Imaging and machine learning in glioblastoma patients”*. 

She described her group’s study that leveraged multiparametric imaging data (anatomic, physiologic, and metabolic) to predict tumor histology with MR-imaging using machine learning. She reported results from tests of several algorithms including random forest, support vector machines, neural networks, and logistic regression for predicting categorical outcomes (tumor score) and random forest (RF), K-nearest neighbor (KNN), linear regression, and stochastic gradient boosting for predicting continuous measures (MIB-1, a cell cycle protein predictive or more aggressive disease and poorer prognosis in astrocytomas). Preliminary results for classification showed that all algorithms perform similarly in terms of misclassification of tumor score as a binary variable (0, ≥ 1). For continuous outcomes, the RF and KNN algorithms performed better than gradient boosting and linear regression. She concluded that no single algorithm works best in situations when down-sampling is used to handle class imbalances. 

## Conclusion 

The 2019 BTEC meeting covered the latest advances in brain tumor epidemiology research and highlighted disparities in brain tumor risk and survival. The next BTEC meeting will be held in Lyon, France, in June 2020. 

## Acknowledgment 

We are thankful for the generous meeting support of the American Brain Tumor Association, the Brain Tumour Foundation of Canada, and the Uncle Kory Foundation. We are also thankful for the tireless dedication of Bénédicte Clement and to Amy Yee who helped coordinate this meeting. 

## Funding 

None. 

## Conflict of interest 

All authors declare that there are no conflicts of interest.
